# Saccadic Adaptation in 10–41 Month-Old Children

**DOI:** 10.3389/fnhum.2016.00241

**Published:** 2016-05-25

**Authors:** Christelle Lemoine-Lardennois, Nadia Alahyane, Coline Tailhefer, Thérèse Collins, Jacqueline Fagard, Karine Doré-Mazars

**Affiliations:** ^1^EA 7326 Laboratoire Vision Action Cognition, Université Paris Descartes, Sorbonne-Paris-CitéBoulogne-Billancourt, France; ^2^CNRS UMR 8242 Laboratoire Psychologie de la Perception, Université Paris Descartes, Sorbonne-Paris-CitéParis, France

**Keywords:** saccadic eye movements, sensori-motor adaptation, motor learning, plasticity, development, infants

## Abstract

When saccade amplitude becomes systematically inaccurate, adaptation mechanisms gradually decrease or increase it until accurate saccade targeting is recovered. Adaptive shortening and adaptive lengthening of saccade amplitude rely on separate mechanisms in adults. When these adaptation mechanisms emerge during development is poorly known except that adaptive shortening processes are functional in children above 8 years of age. Yet, saccades in infants are consistently inaccurate (hypometric) as if adaptation mechanisms were not fully functional in early childhood. Here, we tested reactive saccade adaptation in 10–41 month-old children compared to a group of 20–30 year-old adults. A visual target representing a cartoon character appeared at successive and unpredictable locations 10° apart on a computer screen. During the eye movement toward the target, it systematically stepped in the direction opposite to the saccade to induce an adaptive shortening of saccade amplitude (Experiment 1). In Experiment 2, the target stepped in the same direction as the ongoing saccade to induce an adaptive lengthening of saccade amplitude. In both backward and forward adaptation experiments, saccade adaptation was compared to a control condition where there was no intrasaccadic target step. Analysis of baseline performance revealed both longer saccade reaction times and hypometric saccades in children compared to adults. In both experiments, children on average showed gradual changes in saccade amplitude consistent with the systematic intrasaccadic target steps. Moreover, the amount of amplitude change was similar between children and adults for both backward and forward adaptation. Finally, adaptation abilities in our child group were not related to age. Overall the results suggest that the neural mechanisms underlying reactive saccade adaptation are in place early during development.

## Introduction

Saccadic eye movements constantly redirect the fovea of the retina onto various objects of interest in the environment, allowing optimal visual perception. When saccades are systematically inaccurate, such as after eye muscle paresis (Abel et al., 1978), mechanisms of sensori-motor adaptation come into play to progressively adjust saccade amplitude and reduce saccade endpoint errors. In the laboratory, saccadic adaptation can be induced in few minutes by surreptitiously stepping the target during the saccade (McLaughlin, 1967; for reviews, see Hopp and Fuchs, 2004; Pélisson et al., 2010). Backward intrasaccadic target steps, in the direction opposite to the primary saccade, induce a progressive shortening of saccade amplitude. Forward intrasaccadic target steps, in the same direction as the primary saccade, lead to a lengthening of saccade amplitude. Behavioral and neurophysiological studies in humans and monkeys showed that adaptive shortening and lengthening of saccade amplitude rely on separate mechanisms (Kojima et al., 2004; Catz et al., 2008; Ethier et al., 2008a; Golla et al., 2008; Panouillères et al., 2009).

When these adaptation mechanisms emerge during development is poorly understood. It is generally assumed that adaptation mechanisms compensate for physiological changes such as development. To our knowledge, only two studies have examined adaptation of reactive saccades in children. Salman et al. (2006) found that participants from 8 to 19 years of age show adaptive shortening of saccade amplitude after repetitive backward intrasaccadic target steps. Results from our group (Doré-Mazars et al., 2011) revealed in addition that the amount of saccade amplitude decrease in a group of 11–13 years-old adolescents was similar to adults. These results suggest thus that adaptation mechanisms underlying saccade amplitude shortening are functional from the age of 8 years.

What happens before? This question is relevant for two main reasons. First, infants below 6 months of age consistently make hypometric saccades (Aslin and Salapatek, 1975; Salapatek et al., 1980; Regal et al., 1983; Hainline et al., 1984). We recently showed that saccade hypometria is also a characteristic of babies and toddlers aged from 7 to 42 months compared to adults (Alahyane et al., 2016). However, saccade accuracy is adult-like by 8 years of age (Munoz et al., 1998). It may thus be possible that adaptation processes, at least those that increase saccade amplitude, are not fully functional in the first years of life, thereby leading to more hypometric saccades compared to adults. Second, saccadic adaptation involves a relatively extended brain network including the cerebellum (Takagi et al., 1998; Barash et al., 1999; Straube et al., 2001; Alahyane et al., 2008; Catz et al., 2008; Panouillères et al., 2013, 2015), and cortical parietal and frontal areas (Gaymard et al., 2001; Gerardin et al., 2012; Zimmermann et al., 2015). Important developmental changes in brain structure occur throughout childhood (Pfefferbaum et al., 1994; Matsuzawa et al., 2001; Gogtay et al., 2004; Lenroot and Giedd, 2006; Tiemeier et al., 2010), which may impact saccadic adaptation.

In the present study, we examined both adaptive shortening and lengthening of reactive saccades in young children aged 10–41 months of age compared to a group of adults. We hypothesized that forward adaptation mechanisms underlying saccade amplitude lengthening may not be fully functional in the 10–41 month-old children, leading thus to more hypometric saccades in children compared to adults. Given that forward adaptation and backward adaptation rely on separate mechanisms as we have mentioned previously, we also tested whether children were able to progressively decrease saccade amplitude in response to repetitive backward intrasaccadic target steps. Lower adaptation abilities for both forward and backward adaptation in young children compared to adults would suggest a general functional immaturity of motor learning processes. To induce adaptation in this challenging young population, we developed a protocol in which the saccade targets performed a pseudorandom walk across the computer screen at 10° eccentricity, and adaptation was induced for all tested saccade directions. Moreover, visual stimuli were represented by cartoon characters that were different on every trial. Our protocol allowed to keep children’s interest and motivation for more than a hundred trials, in a relatively short time (less than 15 min), with no specified instruction. Note that we used this protocol, without causing adaptation, to determine the basic characteristics of saccade performance in infants (Alahyane et al., 2016).

## Materials and Methods

### General Methods

Backward adaptation and forward adaptation were tested in two separate experiments. Each experiment was composed of two conditions: an adaptation condition (backward or forward) and a control condition. Adaptation was induced by using the standard double-step target paradigm (McLaughlin, 1967). The control condition was identical to the adaptation condition except that the target never stepped during the saccade. This control condition allowed us to test whether nonspecific factors such as visual experience or fatigue could also affect saccade amplitude (and reaction time). Detailed analysis of basic saccade performance in infants for this control session alone can be found in our recent developmental article (Alahyane et al., 2016). The two adaptation and control conditions were performed at least 3 days apart to avoid any carryover effects between the two sessions (Alahyane and Pélisson, 2005) and the order was randomized across subjects. All experimental procedures were in agreement with the Declaration of Helsinki and were approved by the local institutional ethics committees (CPP no. IRB 00001072). Backward and forward experiments were performed by two independent groups of children recruited from four day-care centers in Paris area. Adults recruited from Paris Descartes University were tested in both backward and forward experiments. Adult participants and children’s parents gave informed and written consent after the nature of the study was fully explained. Adults were tested in the laboratory whereas children were tested in the day-care centers in a dedicated room. The set up and installation were kept as similar as possible between the different test sites.

The general experimental procedure and material are described in detail in our previous article (Alahyane et al., 2016).

#### Apparatus

**Figure 1:**
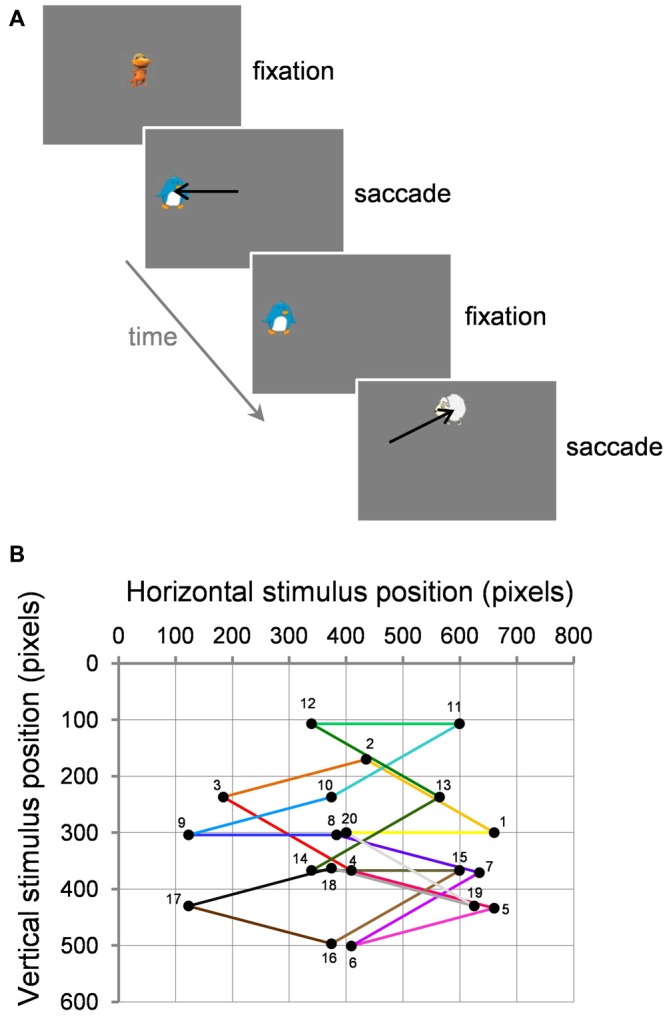
**Task and visual stimuli. (A)** Two example successive trials. **(B)** Example path of 20 successive trials (numbered 1–20) starting from center within a block. Black circles represent the target for the current saccade and the fixation point for the next trial. Note that for adaptation trials, the target stepped during the saccade (not illustrated here; see text for details).

Participants viewed stimuli in a darkened room at a distance of 68 cm. Adults were seated in a chair whereas children were seated on a caregiver’s lap. Stimuli were displayed on a 27 × 36 cm LCD monitor (HM240DT; Iiyama, Nagano, Japan; 800 × 600 pixels resolution at 160 Hz). Stimuli were 1° × 1° colorful cartoon characters used to increase children’s interest and were presented on a medium gray background (Figure 1A). Vision was binocular. The right eye was recorded with the remote Eyelink 1000 eye tracker (SR Research Ltd., Ottawa, ON, Canada) with a sampling rate of 500 Hz and a spatial resolution of 0.05°. A small target sticker was placed on the participants’ forehead to correct eye position measurements across changes in head position.

#### General Procedure

Experiments were programmed with Experimenter Builder (SR Research). We gave no particular instruction about the saccade task. Each experimental session started with a calibration where participants looked at an animated cartoon character appearing sequentially and randomly at five positions over the screen (center, up, down, right, left). The maximal separation between the leftmost target and the rightmost target on the horizontal axis was 27° and the maximal separation between the upper target and the lower target on the vertical axis was 19.1°. This process was renewed to validate that the average error between fixation and target was <1° and that no loss of eye tracking occurred. There was no statistically significant difference in average calibration error between children (0.61° ± 0.52°) and adults (0.55° ± 0.23°; *t*_(113)_ = 0.68; *p* = 0.497; all experimental conditions pooled together).

Each experimental condition (adaptation or control) was divided into seven blocks of 20 trials for the backward condition and into eight 20-trials blocks for the forward condition, with possible rests between blocks. A new stimulus was presented on every trial and became the fixation point of the subsequent trial. For every trial, the experimenter pressed the space bar of the keyboard to validate fixation and proceed to the trial.

In the first trial of each block, an animated stimulus was presented at screen center. Once the participant fixated this stimulus, the experimenter pressed the space bar of the keyboard to validate fixation. The current fixation stimulus disappeared and at the same time a static peripheral target appeared at a location 10° away. The target stayed on the screen until a saccade was detected based on both a 30°/s velocity threshold and a 3000°/s/s acceleration threshold and a boundary criterion: eye position had to cross the boundary of a 4° window centered on target location. As soon as a saccade reached these online criteria, one of two events occurred depending on the type of trial. In the case of control trials where no saccade adaptation was induced, after a delay of 300 ms, the target became animated (shrinking to a size of 0.6° then growing to 3°, shrinking only, or spinning) and a sound (onomatopoeia, musical note) was played. Once the participant successfully fixated the target (which then became the fixation point of the following trial), the experimenter pressed the space bar of the keyboard to validate fixation and proceed to the trial. In the case of adaptation trials, the target stepped in the opposite or same direction as the saccade to induce backward or forward adaptation, respectively. Then, after 300 ms, the target became animated while a sound was played. The stimulus then became the fixation point of the following trial. On every trial, the fixation point also served to perform the standard Eyelink calibration correction to make sure that fixation measurement was still <1°, otherwise calibration was renewed. Over the course of a 20-trial block, the stimuli performed thus a pseudo-random walk across the screen, at 10° eccentricity and along 10 possible directions in both right and left hemifields: on the horizontal 0° axis, up and down at 15° from the horizontal axis, up and down at 30° from the horizontal axis. Each direction was repeated twice within a 20-trial block (Figure 1B).

### Experiment 1: Backward Adaptation

#### Participants

Thirty-nine children (10–42 months-old) participated in the backward adaptation experiment. Among them, five participants (12–34 months-old) did not complete one of the two conditions (adaptation or control) and therefore, their data were discarded from the analyses. Two others did not pass the calibration phase. In sum, we report here on 32 children (age range = 10–41 months; mean ± SD = 29 ± 8.9 months; 14 females). Twenty-six adults (age range = 20–31 years; mean ± SD = 25.5 ± 3.6 years; 21 females) also participated in the backward adaptation experiment. None of the child or adult participants had visual deficits (parental or self-reported) but two adults wore contact lenses and one wore glasses. Twenty-one children and six adults completed the adaptation condition first.

#### Adaptation Condition

The adaptation condition was composed of six 20-trial adaptation blocks (B1 to B6) in which the target stepped in the direction opposite to the primary saccade to induce a progressive shortening of saccade amplitude. In the first three adaptation blocks, the target stepped 2° backward representing a step of 20% relative to original target eccentricity (10°). The last three adaptation blocks contained a backward intrasaccadic step of 3° corresponding thus to 30% of initial target eccentricity. We progressively increased the amplitude of the backward step both to minimize “conscious” detection of the intrasaccadic step by the participants (in particular adults) and to favor a larger adaptation (Alahyane and Pélisson, 2005). These adaptation blocks were preceded with a pre-adaptation block of 20 trials (block B0) where the target did not step during saccade execution. These pre-adaptation trials were equivalent to control trials and provided baseline measures of saccade performance before adaptation.

#### Control Condition

The design and the number of both blocks and trials were identical to the adaptation condition described above. The only difference was that backward intrasaccadic target steps were never introduced here.

### Experiment 2: Forward Adaptation

#### Participants

Forty-one children (age range = 10–38 months; mean ± SD = 28 ± 6.4 months; 17 females) were recruited for the forward experiment and completed both adaptation and control sessions. None had deficits in vision or wore correction glasses. A group of 17 adults (age range = 21–31 years; mean ± SD = 26 ± 3.5 years; 16 females) was included for comparison. All of these adults had participated in the backward experiment a year before. One adult wore glasses and one wore contact lenses. Twenty-one children and three adults completed the adaptation condition first.

#### Adaptation Condition

The adaptation condition was divided into seven 20-trial adaptation blocks (B1 to B7) in which the target stepped in the same direction as the primary saccade to induce a progressive lengthening of saccade amplitude. Note that we added a block of 20 adaptation trials compared to the backward experiment because of the well-known slower rate for forward compared to backward adaptation (Pélisson et al., 2010). Here again, the amplitude of the intrasaccadic target step increased across blocks of trials to minimize their “conscious” perception and induce a larger adaptation. The forward intrasaccadic step represented 10% of the original target eccentricity (10°) in the first two adaptation blocks, 20% in the next two blocks and 30% in the last three adaptation blocks. These seven adaptation blocks were preceded by a pre-adaptation block (block B0) where the target did not step during the saccade, providing measures of baseline saccade performance before adaptation.

#### Control Condition

The design and length of the control session were identical to the forward adaptation session described above except that forward intrasaccadic target steps were never introduced.

### Data Analyses

Data were processed with Data Viewer (SR Research) and Microsoft Excel while statistical analyses were done with Statistica (Statsoft).

We analyzed the first, or primary, saccade after target appearance on every trial. All saccade directions were pooled together as their effect was not the purpose of the study, and there were too few trials per direction. Saccade reaction time (SRT) was defined as the time interval between fixation point disappearance and saccade onset. Saccade amplitude was computed as the difference between the final and initial eye position.

We discarded trials with blinks contaminating the saccade, SRT shorter than 50 ms or longer than 600 ms, saccade amplitude shorter than 4° or longer than 16°, saccade direction outside an angle of 30° around target position, and outliers. Outliers were values that lied below (Q1−2.3×IQR) or above (Q3 + 2.3×IQR), Q1 and Q3 being the first and third quartiles respectively and IQR the interquartile range (Tukey box plot; Carling, 2000). For the backward experiment (adaptation and control conditions pooled together), the proportion of discarded trials for children and adults, respectively, was: 5.2% and 4.1% for blinks, 5.6% and 9.5% for incorrect directions, 0% and 0.1% for SRTs < 50 ms, 0.3% and 0.1% for SRTs > 600 ms, 0.5% and 0.6% for amplitudes < 4°, 0.2% and 0.1% for amplitudes > 16°, 3.9% and 4.6% for outliers. Note that a few other trials in children were contaminated by eye recording loss and were thus discarded (1.4%). For the forward experiment (adaptation and control conditions pooled together), the proportion of discarded trials for children and adults, respectively, was: 5.7% and 6.2% for blinks, 6.4% and 8.8% for incorrect directions, 0% and 0.08% for SRTs < 50 ms, 0.7% and 0.1% for SRTs > 600 ms, 0.7% and 0.3% for amplitudes < 4°, 0.2% and 0.2% for amplitudes > 16°, 4.2% and 3.9% for outliers. A few other trials contaminated by eye tracking loss in children were also discarded (1.4%). Based on all these exclusion criteria, the proportion of valid trials for the backward experiment was 83 ± 1% for children and 81 ± 1% for adults. Each child contributed to 78–134 scorable trials and each adult contributed to 79–132 trials. For the forward experiment, the proportion of valid trials was 80 ± 1% for children and 80 ± 2% for adults. Each child contributed to 85–149 valid trials and each adult contributed to 91–149 trials. The proportion of valid trials was similar between control and adaptation conditions for each backward and forward experiment and for each age group. Importantly, the similar proportions of scorable trials between children and adults (unpaired *t*-tests, *p*s > 0.23) precluded any effect of number of trials.

The backward and forward experiments were analyzed separately. Note that in each of these experiments and in both adult and child groups, there was no difference in saccade baseline amplitude (block B0) between the subjects who performed the adaptation condition first and those who performed the control condition first (*t*-tests for independent samples, *p*s > 0.08). Saccade amplitude was compared across blocks of trials, for the adaptation and control conditions separately, by repeated measures ANOVAs with Blocks of trials as a within-subject factor and Age group as a between-subject factor. They were followed by *post hoc* Tukey high significance difference (HSD) tests. Similar analyses were performed for saccade reaction time to examine a possible effect of fatigue or strategy over the course of the experiment. The effect of exposure of repetitive intrasaccadic target steps was also estimated at the individual level by *t*-tests for independent samples that compared saccade baseline amplitude at block B0 and saccade amplitude in the last adaptation block.

The amount of adaptation (%) was calculated classically using the following formula:

Percent amplitude change = [(mean amplitude in the last adaptation block−mean amplitude in block B0)/mean amplitude in B0] × 100.

Developmental effects were assessed by comparing saccade baseline performance (amplitude, reaction time) and the amount of adaptation between adults and children with *t*-tests for independent samples. We also performed correlations between the percent amplitude change and age within the child group.

Significance was set at alpha = 0.05. Note that there was no significant effect of gender for any of the analyses (*p*s > 0.05).

## Results

### Experiment 1: Backward Adaptation

#### Baseline Performance

Repeated measures ANOVAs with condition (adaptation vs. control) as a within-subject factor and age group (children vs. adults) as a between-subject factor were performed on baseline SRT and baseline saccade amplitude (i.e., in block B0) separately. There was only a significant effect of Age group for both SRT (*F*_(1,56)_ = 91.4, *p* < 0.0001) and saccade amplitude (*F*_(1,56)_ = 48, *p* < 0.0001). Children showed on average longer SRT (235 ± 6 ms) than adults (163 ± 3 ms), and their saccades were more hypometric (7.93 ± 0.08°) than adults’ (8.85 ± 0.11°), in accordance with our previous study (Alahyane et al., 2016).

#### Adaptation Condition

**Figure 2:**
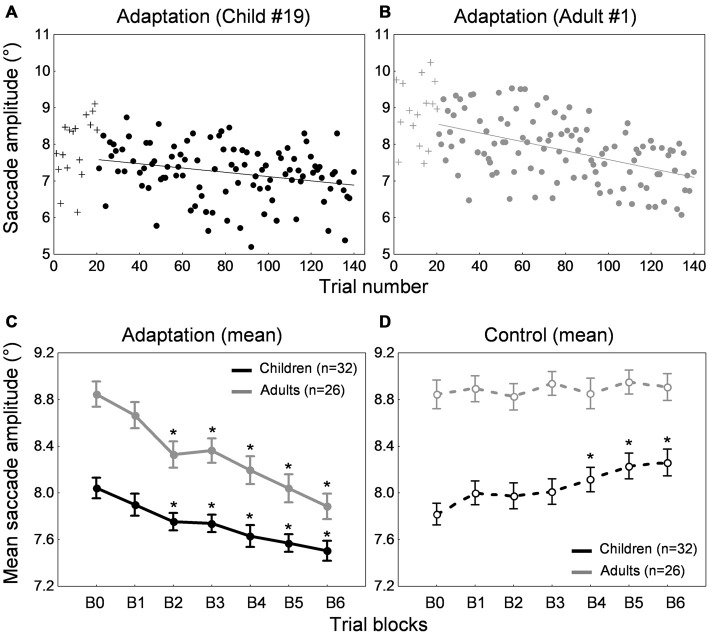
**Backward adaptation experiment. (A,B)** Time course of saccade amplitude as a function of trial number in one representative child (30 months-old) and one representative adult (20 years-old) in the adaptation condition. Crosses represent data in the preadaptation 20-trial-block B0. Filled circles represent data in the adaptation blocks. **(C)** Mean saccade amplitude as a function of blocks of trials in the child group (black trace) and the adult group (gray trace) in the adaptation condition. Block B0 corresponds to the preadaptation block with no intrasaccadic target step, blocks B1 to B3 to adaptation blocks with an intrasaccadic step of 20% relative to initial target eccentricity, blocks B4 to B6 to adaptation blocks with an intrasaccadic step of 30%. **(D)** Mean saccade amplitude as a function of blocks of trials in the child group (black trace) and the adult group (gray trace) in the control condition. The seven blocks of trials were similar to the adaptation condition except that there were never intrasaccadic target steps. **(C,D)** Asterisks illustrate statistically significant differences compared to the first block B0 (*post hoc* Tukey HSD tests, *p*s < 0.01); others: ns. Errors bars represent SEM.

**Figure 3:**
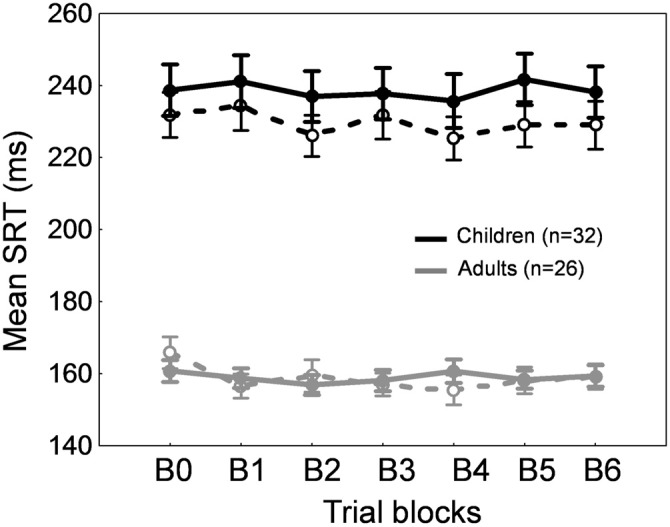
**Backward adaptation experiment**. Mean saccade reaction time across blocks of trials in children (black traces) and adults (gray traces) for the adaptation condition (solid lines) and the control condition (dashed lines). Errors bars are SEM.

Figures 2A,B show the time course of saccade amplitude as a function of trial number in one representative child (30 months of age) and one representative adult (20 years of age). Similar to the adult, the child showed a progressive decrease in saccade amplitude across adaptation trials, consistent with the backward intrasaccadic target step introduced from trial #21 (see “Materials and Methods” Section). As illustrated in Figure 2C, at the group level, saccade amplitude gradually decreased across the six 20-trial-adaptation blocks in both adult and child groups compared to the first, preadaptation, block B0. A repeated measures ANOVA revealed significant main effects of Blocks of trials (*F*_(6,336)_ = 49.6, *p* < 0.0001) and Age group (*F*_(1,56)_ = 24.9, *p* < 0.0001), and a significant interaction effect (*F*_(6,336)_ = 4.14, *p* < 0.001). *Post hoc* Tukey HSD tests indicated that mean saccade amplitude was shorter in children than in adults for all blocks (*p*s < 0.05) except B5 and B6 (*p*s > 0.057) and importantly, mean saccade amplitude in blocks B2–B6 was lower than in block B0 (*p*s < 0.01) in both age groups, illustrating significant adaptation. This shortening of saccade amplitude was not due to strategy or fatigue because there was no change in SRT across blocks of trials in children or adults (Figure 3, solid lines). Indeed we found only a significant effect of Age group (*F*_(1,56)_ = 95.5, *p* < 0.0001), children showing longer SRT than adults, but no significant effect of Blocks of trials or interaction (*p*s > 0.2).

**Figure 4:**
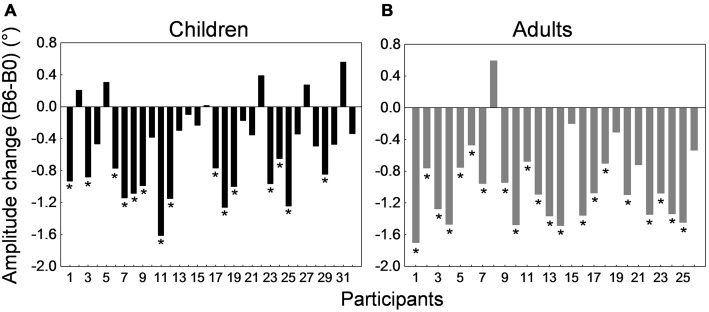
**Backward adaptation experiment.** Saccade amplitude change measured by the difference between mean saccade amplitude in block B6 and the mean amplitude in block B0 for all 32 children **(A)** and all 26 adults **(B)**. Note that in both panels participants were arranged by ascending age to facilitate comparisons. For the child group, the age range was 10–16 months for participants #1 to #4, 20–25 months for participants #5 to #11, 26–30 months for participants #12 to #19, 35–37 months for participants #20 to #25, and 38–41 months for participants #26 to #32. In both panels, asterisks depict statistically significant differences in amplitude between blocks B6 and B0 (*t*-tests, *p*s < 0.05). Note that data from child #19 and adult #1 were used to illustrate Figures 2A,B.

Figure 4 depicts the amplitude change between the preadaptation block B0 and the last adaptation block B6 for all 32 children and 26 adults. Interestingly, most participants showed a decrease in saccade amplitude. This decrease was significant in 21 of the 26 adults (representing a proportion of 81%) and in 15 of the 32 children (i.e., 47%; *p*s < 0.05; *t*-tests for independent samples between blocks B0 and B6). When we compared the children who exhibited a significant shortening of amplitude and children who did not show significant adaptation, we found that they did not differ in age (*t*_(30)_ = −1.31, *p* = 0.2) but did differ in saccade baseline amplitude in the preadaptation block (*t*_(30)_ = 2.51, *p* < 0.05). In other words, children who did not adapt significantly were those who made more hypometric saccades at the beginning of the experiment.

#### Control Condition

We also ran a control condition, where no backward intrasaccadic target step was introduced, to examine whether some unspecific factors (e.g., fatigue, training) may contribute to the effects seen in the adaptation condition. While saccade amplitude was stable across blocks of trials in adults, saccade amplitude progressively increased in the child sample (Figure 2D), replicating our previous study (Alahyane et al., 2016). These trends were confirmed by a repeated measures ANOVA showing an interaction between the within-subject factor “blocks of trials” and the between-subject factor “age group” (*F*_(6,336)_ = 3.28, *p* < 0.005). Children showed larger saccade amplitude in blocks B4–B6 compared to B0 (*p*s < 0.002) while no differences were found in adults (*post hoc* Tukey tests).

Concerning SRT (Figure 3, dashed lines), we found a significant Blocks × Age group interaction (*F*_(1,336)_ = 2.2, *p* < 0.05). SRT was longer in children than in adults for all blocks as expected (*post hoc* tests, *p*s < 0.001) and importantly, SRT was stable across blocks of trials in both children and adults except a shorter SRT in B4 compared to B0 in adults (*p* < 0.05).

Thus the decrease in saccade amplitude across trials in the adaptation condition for both adults and children was related to the systematic backward intrasaccadic target steps rather than unspecific factors.

#### Amount of Adaptation

We next quantified the percent amplitude change between the preadaptation block B0 and the last adaptation block B6 (see “Materials and Methods” Section). Adults exhibited an amplitude decrease of 10.8 ± 1.1% while children showed a decrease of 6.48 ± 1.2%. However, results in the control condition (Figure 2D) highlighted an important difference in children compared to adults. Contrary to adults, saccade amplitude gradually increased across blocks of trials in children. This suggests that in children, the baseline changed over the course of the experimental session. Thus, calculating a percentage of amplitude change in the adaptation condition between the last adaptation block and the preadaptation block, as it is classically done in adults, was not appropriate in children. Instead we used the last control block B6 as a baseline to compute the amount of adaptation as follows:

Percent amplitude change = [(mean amplitude in the last adaptation block B6 – mean amplitude in the last control block B6)/mean amplitude in the last control block B6] × 100.

**Figure 5:**
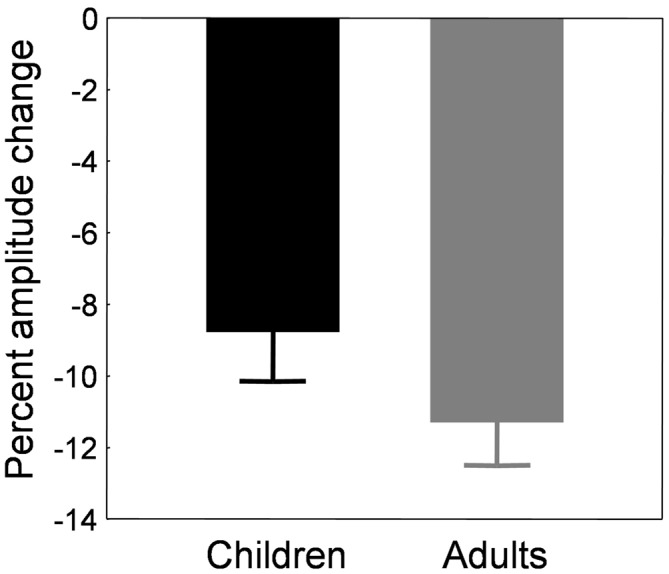
**Backward adaptation experiment.** Mean percent amplitude change in children (black bars) and adults (gray bars) in the adaptation condition. The baseline used to compute the amount of adaptation was the last control block B6 instead of the preadaptation block B0. See text for rationale and details.

Note that using this new formula or the classical formula in adults did not affect the results, which was expected given that saccade amplitude was stable across blocks of trials in the control condition (Figure 2D, gray trace). Figure 5 illustrates the mean percent amplitude change in both child and adult groups using this new formula (i.e., using the last control block as a baseline to compute the percent amplitude change). The reduction of saccade amplitude in the child sample reached 8.8 ± 1.4% on average. The percent amplitude change did not correlate with age (*r* = 0.044, *p* = 0.812). Adults exhibited an amplitude decrease of 11.3 ± 1.2%, an amount that did not differ significantly from children’s (*t*_(56)_ = 1.35, *p* = 0.182).

This first experiment revealed that our adaptation protocol successfully led to significant shortening of saccade amplitude not only in adults but also in children. Thus backward adaptation mechanisms are functional in 10–41 month-old children. What about forward adaptation? We predicted that the pronounced saccade hypometria in children may reflect a selective deficit of forward adaptation mechanisms that may not be functional as early as backward adaptation processes. We next investigated forward adaptation in a group of 41 children (10–38 months-old) compared to a group of 17 adults (21–31 years-old; see “Materials and Methods” Section).

### Experiment 2: Forward Adaptation

#### Baseline Performance

SRT and saccade amplitude in block B0 were submitted separately to repeated measures ANOVAs with condition (adaptation vs. control) as a within-subject factor and age group (children vs. adults) as a between-subject factor. We only observed a significant effect of Age group for both SRT (*F*_(1,56)_ = 79.9, *p* < 0.0001) and saccade amplitude (*F*_(1,56)_ = 42.9, *p* < 0.0001). As expected, children showed on average longer SRT (231 ± 5 ms) than adults (157 ± 3 ms), and their saccades were more hypometric (8.05 ± 0.08°) than adults’ (8.97 ± 0.09°).

#### Adaptation Condition

**Figure 6:**
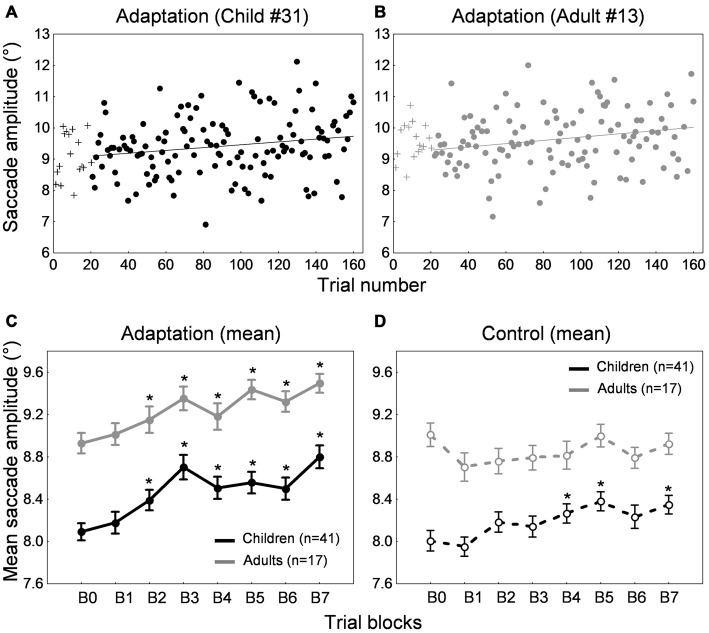
**Forward adaptation experiment. (A,B)** Time course of saccade amplitude as a function of trial number in one representative child (33 months-old) and one representative adult (28 years-old) in the adaptation condition. Crosses represent data in the preadaptation 20-trial-block B0. Filled circles represent data on adaptation trials. **(C)** Mean saccade amplitude as a function of blocks of trials in the child group (black trace) and the adult group (gray trace) in the adaptation condition. Block B0 corresponds to the preadaptation block with no intrasaccadic target step, blocks B1 and B2 to adaptation blocks with an intrasaccadic step of 10% relative to initial target eccentricity, blocks B3 and B4 to adaptation blocks with an intrasaccadic step of 20% and blocks B5 to B7 to adaptation blocks with an intrasaccadic step of 30%. **(D)** Mean saccade amplitude as a function of blocks of trials in the child group (black trace) and the adult group (gray trace) in the control condition. The eight blocks of trials were similar to the adaptation condition except that there were never intrasaccadic target steps. **(C,D)** Asterisks illustrate statistically significant differences compared to the first block B0 (*post hoc* Tukey HSD tests following a significant effect of Block of trials factor in **(C)**, *p*s < 0.0001; *post hoc* Tukey HSD tests following a significant Blocks of trials × Age group interaction in **(D)**, *p*s < 0.05); others: ns. Errors bars represent SEM.

Figures 6A,B shows the time course of saccade amplitude as a function of trial number in one representative child (33 months of age) and one representative adult (28 years of age). Both child and adult exhibited a progressive increase in saccade amplitude across adaptation trials, consistent with the forward intrasaccadic target step introduced from trial #21 (see “Materials and Methods” Section). As illustrated in Figure 6C, at the group level, saccade amplitude gradually increased across the seven 20-trial-adaptation blocks in both adult and child groups compared to the first, preadaptation, block B0. A repeated measures ANOVA revealed significant main effects of Blocks of trials (*F*_(7,392)_ = 23.3, *p* < 0.0001) and Age group (*F*_(1,56)_ = 25.7, *p* < 0.0001), but no significant interaction effect (*F* < 1). *Post hoc* Tukey HSD tests indicated that mean saccade amplitude in blocks B2–B6 was larger than in block B0 (*p*s < 0.0001), illustrating significant adaptation in both age groups.

**Figure 7:**
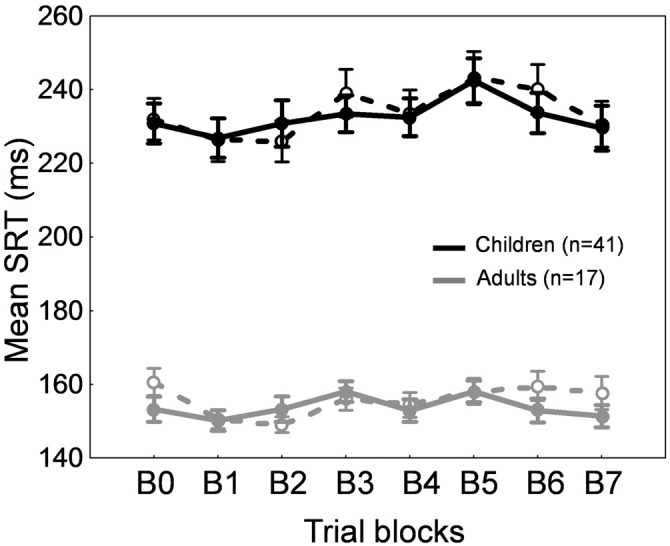
**Forward adaptation experiment.** Mean saccade reaction time across blocks of trials in children (black traces) and adults (gray traces) for the adaptation condition (solid lines) and the control condition (dashed lines). Errors bars are SEM.

A repeated measures ANOVA performed on SRT also showed significant effects of Blocks of trials (*F*_(7,392)_ = 4.91, *p* < 0.0001) and Age group (*F*_(1,56)_ = 86.5, *p* < 0.0001) factors, but no interaction (*F* < 1). SRT in block B5 only was significantly higher than SRT in block B0 (*p* < 0.001). This lack of consistent changes in SRT across blocks of trials (Figure 7, solid lines) suggests that the increase in saccade amplitude (Figure 6C) was not related to strategy or fatigue.

**Figure 8:**
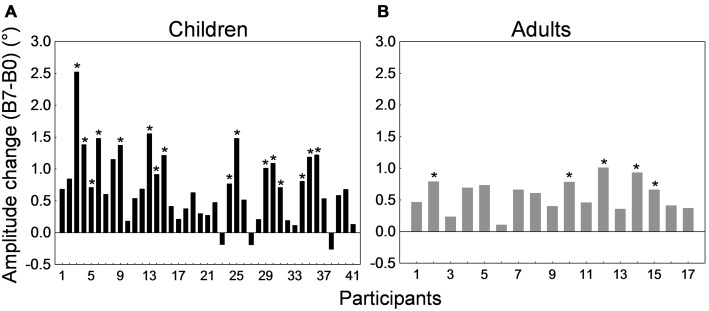
**Forward adaptation experiment.** Saccade amplitude change measured by the difference between mean saccade amplitude in block B7 and the mean amplitude in block B0 for all 41 children **(A)** and all 17 adults **(B)**. Note that in both panels participants were arranged by ascending age to facilitate comparisons. For the child group, the age range was 10–19 months for participants #1 to #3, 21–25 months for participants #4 to #10, 26–29 months for participants #11 to #25, 30–35 months for participants #26 to #35, and 36–38 months for participants #36 to #41. In both panels, asterisks depict statistically significant differences in amplitude between blocks B7 and B0 (*t*-tests, *p*s < 0.05). Note that data from child #31 and adult #13 were used to illustrate Figures 6A,B.

Figure 8 depicts the amplitude change between the preadaptation block B0 and the last adaptation block B7 for all 41 children and 17 adults. All adults and 38 children showed larger saccade amplitude in block B7 than B0. *T*-tests for independent samples between B0 and B7 performed for each subject revealed that this increase in amplitude was significant in 5 of the 17 adults and in 16 of the 41 children (*p*s < 0.05). Comparison of child participants exhibiting significant vs. no significant amplitude change revealed no difference in age or in baseline amplitude (*t*-tests for independent samples, *p*s > 0.19).

#### Control Condition

In the control condition that contained no forward intrasaccadic target steps, we again found a progressive increase in saccade amplitude in children but no modification of amplitude across blocks of trials in adults (Figure 6D). A repeated measures ANOVA showed a significant interaction between the within-subject factor “blocks of trials” and the between-subject factor “age group” (*F*_(7,392)_ = 2.69, *p* < 0.01). Children showed larger saccade amplitude in blocks B4–B5 and B7 compared to B0 (*p*s < 0.05) while no differences were found in adults (*post hoc* Tukey HSD tests).

For SRT, we observed a significant effect of Blocks of trials (*F*_(7,392)_ = 7.91, *p* < 0.0001) and of Age group (*F*_(1,56)_ = 67.014, *p* < 0.0001). SRT was longer in children than in adults for all blocks as expected (*post hoc* tests, *p*s < 0.001). Compared to block B0, SRT was significantly lower in blocks B1 and B2, and higher in block B5 (*p*s < 0.05). This absence of consistent changes in SRT across blocks of trials (Figure 7, dashed lines) rules out any effect of strategy or fatigue.

#### Amount of Adaptation

**Figure 9:**
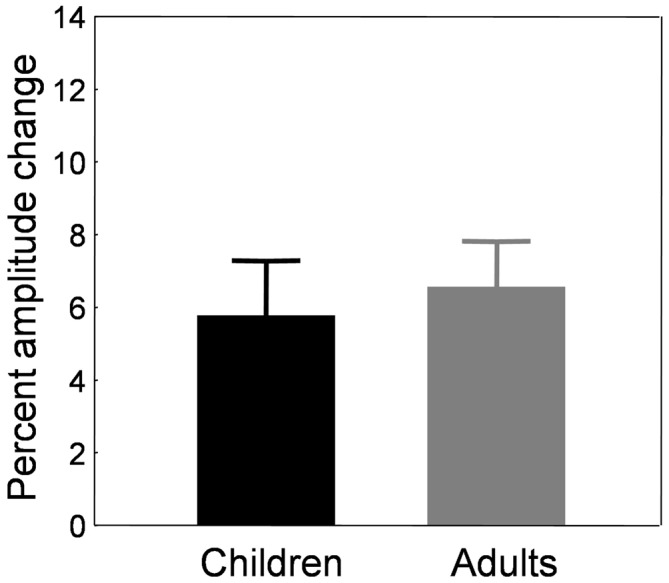
**Forward adaptation experiment.** Mean percent amplitude change in children (black bars) and adults (gray bars) in the adaptation condition. The baseline used to compute the amount of adaptation was the last control block B7 instead of the preadaptation block B0. See text for rationale and details.

The amount of adaptation between the preadaptation block B0 and the last adaptation block B7 represented 6.41 ± 0.71% in adults and 8.88 ± 1.12% in children. However, here again, the increase in saccade amplitude in the control condition in children suggests that the baseline changes over the course of the experiment. Similar to the backward adaptation condition above, we used the last control block (B7) as the baseline block instead of the preadaptation block B0 to compute the amount of adaptation, for both children and adults. These amounts of adaptation are illustrated in Figure 9.

Children showed an amplitude increase of 5.77 ± 1.51%. The percent amplitude change did not correlate with age (*r* = −0.168, *p* = 0.292). Adults exhibited an amplitude increase of 6.56 ± 1.26%, an amount that was not statistically different from that of the children (*t*_(56)_ = −0.318, *p* = 0.751).

#### Comparison to Backward Adaptation

We tested differences between the backward and forward adaptation conditions by comparing the amount of adaptation depicted in Figures 5, 9 with *t*-tests for independent samples. In adults, the amplitude change was higher in the backward condition (*n* = 26; 11.3 ± 1.2%) than the forward condition (*n* = 17; 6.56 ± 1.26%; *t*_(41)_ = 2.64, *p* < 0.05). A similar trend was found in children but the difference between backward (*n* = 32; 8.8 ± 1.4%) and forward (*n* = 41; 5.77 ± 1.51%) adaptation was not significant (*t*_(71)_ = 1.43, *p* = 0.156). In sum, the backward condition induced greater change than the forward condition, as classically described in the literature for standard adaptation protocols in which one saccade vector was adapted (for review, see Pélisson et al., 2010).

This second experiment demonstrated that our adaptation protocol led to significant lengthening of saccade amplitude in both adults and children. Thus forward adaptation mechanisms are also functional in young children from 10 months of age.

## Discussion

The goal of our study was to test whether saccade hypometria which is consistently found in infants and toddlers could be related to a deficit of forward adaptation mechanisms that lengthen saccade amplitude. Contrary to our hypothesis, we found that children from 10 to 41 months of age were able to adapt to systematic forward intrasaccadic target steps. They also showed a gradual shortening of saccade amplitude in response to repetitive backward intrasaccadic target steps. Saccadic adaptation was not influenced by age in our child sample. These results show thus that reactive saccade adaptation processes are in place in the 10–41 month-olds.

### Saccadic Adaptation Paradigm in Children

Using an appropriate adaptation protocol in young children was a first challenge to cope with because we needed a sufficient number of trials to induce adaptation while keeping the experiment motivating and the shortest possible in duration. We thus developed an original protocol where visual targets were represented by colorful cartoon characters performing a pseudo-random walk across the screen at 10° eccentricity, and adaptation was induced for all 10 saccade directions. Moreover, the target was novel at every trial. But this did not *a priori* impact saccadic adaptation as it has been shown that saccade adaptation can be induced with complex targets and it does not depend on target shape or color (Deubel, 1995; Bahcall and Kowler, 2000; Azadi and Harwood, 2014). This experimental protocol was successful because it allowed us to maintain children’s interest over 140 or 160 trials (in the backward or forward condition, respectively), in less than 15 min, with no specified instruction. Importantly, 73 of the 78 children completed both adaptation and control conditions on separate days.

Our protocol differs from standard adaptation protocols that usually test simple saccade targets and adapt only saccades with one amplitude and one direction in adults. Nevertheless we successfully induced saccadic adaptation in adults. Indeed, adults showed significant saccade amplitude shortening or lengthening consistent with the repetitive intrasaccadic target step (backward or forward, respectively) whereas no change in saccade amplitude was observed in the control condition in which there were no intrasaccadic target steps. No change in SRT was shown across blocks of trials, excluding any effect of fatigue or strategy. Finally, the amount of backward adaptation was significantly greater than the amount of forward adaptation, in agreement with previous studies using standard adaptation protocols (for review see Pélisson et al., 2010). Another study in adults tested backward adaptation of saccades performed toward 0.2°-diameter dots stepping in a pseudo-random walk across the screen (Rolfs et al., 2010). Their protocol differed from ours since the target steps could be in any direction between 0° and 359° and with an amplitude range of 4–12°. The amount of adaptation in their study (12.5%) was however close to ours (11.3%).

For comparison, we also ran in adults a more conventional adaptation experiment (not shown) in which we induced adaptation of reactive saccades directed toward a visual target always appearing on the right of the fixation point, at 10° eccentricity. All other aspects of the procedure of this “one saccade vector” adaptation experiment were the same as the “multiple saccade vectors” adaptation experiment described and analyzed in the present study. We found that the change in saccade amplitude between the pre-adaptation block and the last adaptation block was larger in the “one saccade vector” experiment than in the “multiple saccade vectors” experiment (amplitude decrease of 16.8 ± 1.14% vs. 10.8 ± 1.1% for backward adaptation; amplitude increase of 13.3 ± 2.2% vs. 6.41 ± 0.71% for forward adaptation; see also Scudder et al., 1998). These results were expected as a same saccade vector (10° amplitude, horizontal rightward direction) was “trained” 120 times (or 140 times) in the “one-vector” experiment for the backward (or forward) adaptation condition. In contrast, in the present study, 10 saccade directions were tested and each one was trained 12 or 14 times over the course of the backward or forward adaptation condition, respectively. These complementary results confirm the well-known vector-specificity of saccadic adaptation in monkeys (Straube et al., 1997; Noto et al., 1999) and humans (Deubel, 1987; Frens and van Opstal, 1994; Collins et al., 2007; Alahyane et al., 2008). Our “multiple saccade vectors” protocol, however, induced significant adaptation in adults suggesting that it provides a good and rapid model to study saccadic adaptation in young children.

### Adaptation Mechanisms for Reactive Saccades are in Place in Young Children

Previous studies revealed that adaptation mechanisms underlying saccade amplitude shortening are functional in children above 8 years of age (Salman et al., 2006; Doré-Mazars et al., 2011). Here, we found that both these adaptation mechanisms and those underlying saccade amplitude lengthening are in place well before, during the first years of life. Similar to adults, children in the age range of 10–41 months exhibited saccade amplitude changes consistent with the direction (backward or forward) of the repetitive intrasaccadic target steps. Moreover, they showed similar amount of adaptation to adults for both backward and forward conditions. Finally, adaptation abilities were not related to age in the child sample. Behavioral, neurophysiological, imaging and lesion studies proposed that reactive saccade adaptation mechanisms involve the final stage of oculomotor processing including the cerebellum vermis and the superior colliculus or the brainstem (Frens and van Opstal, 1994; Melis and van Gisbergen, 1996; Desmurget et al., 1998; Takagi et al., 1998; Barash et al., 1999; Edelman and Goldberg, 2002; Alahyane et al., 2004, 2007, 2008; Takeichi et al., 2007; Catz et al., 2008; Golla et al., 2008; Panouillères et al., 2009, 2013, 2015; Xu-Wilson et al., 2009; Kojima et al., 2010; Gerardin et al., 2012). While the brain continues to undergo structural changes in gray matter and white matter through childhood and adolescence (for reviews see Johnson, 2001; Luna et al., 2008), our study suggests that the basic neural circuitry involved in sensori-motor adaptation is in place in early childhood. Nevertheless, this should not be automatically extended to voluntary saccades. Adaptation mechanisms are partially separate between reactive and voluntary saccades (for review, see Pélisson et al., 2010). In particular, it is suggested that voluntary saccade adaptation relies on early stages of oculomotor processing (involving notably parietal and frontal cortical areas) and the lateral cerebellum (Deubel, 1995; Alahyane et al., 2007, 2008; Cotti et al., 2007; Gerardin et al., 2012; Panouillères et al., 2013). These brain regions are still maturing during childhood, and even adolescence (Gogtay et al., 2004; Lenroot and Giedd, 2006; Tiemeier et al., 2010). It is thus possible that, contrary to reactive saccade adaptation, voluntary saccade adaptation may not be functional early during development. We have begun new experiments in our lab to explore this exciting hypothesis.

An interesting point in our study was that not only did the children show efficient forward adaptation contrary to our initial hypothesis but their adaptation abilities were not related to their initial saccade hypometria. In other words, children who made more hypometric saccades did not show larger saccade amplitude increase in response to the forward intrasaccadic target steps. It is tempting to speculate that, similar to adults (Bahcall and Kowler, 2000; Collins and Wallman, 2012), the error signal leading to adaptive saccade lengthening may not be simply a post-saccadic visual error in children. Indeed, the amount of forward adaptation would have been larger in children generating shorter saccades, and even larger than the backward adaptation, which was not the case here. Note that we observed that children who did not show significant backward adaptation were those who made more hypometric saccades. But this was not surprising given that saccades in these children may have landed closer to the final position of the target and could not be shortened further. In sum, similar to adults, saccade hypometria may be favored in young children, perhaps to minimize total saccadic flight time (Harris, 1995) or to allow the generation of primary and secondary saccades from the same brain hemisphere (Robinson, 1973).

At last, in agreement with our past results (Alahyane et al., 2016), we found that children also showed a progressive increase in saccade amplitude across blocks of trials in the control condition where no intrasaccadic target steps were introduced. This increase in amplitude may be related to visual experience and/or sensori-motor learning. Whether the saccade amplitude lengthening observed in this control condition and the saccade lengthening induced by exposure to systematic artificial errors rely on similar or separate processes needs further investigations.

### Why are Saccades Hypometric in Children?

We showed that sensori-motor learning processes are functional in young children and they can lead to a lengthening of saccade amplitude when necessary. Then, why do children consistently generate more hypometric saccades than adults?

One possibility may be that their saccade hypometria may be related to immature peripheral neural systems. In particular, if the fovea becomes adult-like by 15–18 months of age, it continues to mature until 4–5 years of age (Hendrickson and Yuodelis, 1984; Hendrickson, 1992; Dubis et al., 2012). However, if this were the only reason for saccade inaccuracy in young children, then saccade adaptation should be influenced by age, which was not the case here.

Another attractive hypothesis could be related to adaptation retention. It has been proposed that saccadic adaptation is supported by two processes: a fast adaptation process that learns quickly from errors but is subjected to forgetting, and a slow process that learns slowly from errors but shows strong retention (Ethier et al., 2008b; Xu-Wilson et al., 2009). We can thus suppose that the slow and enduring adaptation process is not fully functional in children contrary to the rapid and “more labile” adaptation process. In other words, even if children are able to progressively recover saccade accuracy, this learning is not retained. However this does not mean that exposure to repetitive intrasaccadic target steps in the laboratory does not lead to true plastic changes. Indeed, significant saccade gain changes in adults were still observed three or five few days after the adaptation experiment, and even longer (Alahyane and Pélisson, 2005; Wang et al., 2012). Unfortunately our experimental protocol did not include a postadaptation block of trials with no visual feedback to measure the after-effect of saccadic adaptation, which would have provided some first cues on retention in young children. Future studies are thus necessary to address this issue.

In conclusion, our study shows that reactive saccade adaptation processes are in place early during development. But this is hopefully only the beginning of a new and exciting research area on the acquisition, development and consolidation of sensori-motor learning processes that ensure the maintenance of saccade accuracy and optimal vision through the lifespan.

## Author Contributions

CL-L, NA, TC, JF and KD-M contributed to the conception and design of the experiments. CL-L, NA and CT ran the experiments. CL-L and NA analyzed the data. All authors discussed on the results and their interpretation. NA wrote the article. CL-L, TC, and KD-M edited the article. All authors approved the final version of the article.

## Conflict of Interest Statement

The authors declare that the research was conducted in the absence of any commercial or financial relationships that could be construed as a potential conflict of interest.
